# Therapeutic potential of mGluR5 targeting in Alzheimer's disease

**DOI:** 10.3389/fnins.2015.00215

**Published:** 2015-06-09

**Authors:** Anil Kumar, Dinesh K. Dhull, Pooja S. Mishra

**Affiliations:** ^1^UGC Centre of Advanced Studies, University Institute of Pharmaceutical Sciences, Panjab UniversityChandigarh, India; ^2^Department of Neurophysiology, National Institute of Mental Health and Neuro SciencesBangalore, India

**Keywords:** Alzheimer's disease, MPEP, amyloid-β, glia, mGluR, neurodegeneration

## Abstract

Decades of research dedicated toward Alzheimer's disease (AD) has culminated in much of the current understanding of the neurodegeneration associated with disease. However, delineating the pathophysiology and finding a possible cure for the disease is still wanting. This is in part due to the lack of knowledge pertaining to the connecting link between neurodegenerative and neuroinflammatory pathways. Consequently, the inefficacy and ill-effects of the drugs currently available for AD encourage the need for alternative and safe therapeutic intervention. In this review we highlight the potential of mGluR5, a metabotropic glutamatergic receptor, in understanding the mechanism underlying the neuronal death and neuroinflammation in AD. We also discuss the role of mGlu5 receptor in mediating the neuron-glia interaction in the disease. Finally, we discuss the potential of mGluR5 as target for treating AD.

## Introduction

Alzheimer's disease (AD) is a progressive neurodegenerative disorder characterized by memory loss, cognitive impairment, and changes in behavior and personality (Reddy and McWeeney, 2006). The disease continues to be a major public health issue owing to its increasing prevalence, long duration, high cost of care, and lack of disease-modifying drugs Deaths due to AD have been rising dramatically compared to other life compromised diseases. According to the survey conducted for the period between 2000 and 2008 (preliminary data), while there was a reduction in the deaths due to heart diseases by 13%, stroke by 20%, and prostate cancer by 8%, the deaths because of AD increased by 66% (Alzheimer's Association, 2011).

Although the pathology of AD was described more than 100 years ago, its research has gained momentum only in the last 3 decades. Moreover, despite the extensive research, none of the currently available drug offers an effective treatment. Therefore, the need arises to understand disease pathogenesis, identify novel targets and develop effective clinical treatment.

The neuropathology of AD is characterized by the presence of extracellular senile plaques and intracellular neurofibrillary tangles composed of amyloid-β (Aβ), a cleavage product of the amyloid precursor protein; and aberrantly phosphorylated tau (Glenner and Wong, 1984; Price and Sisodia, 1998). Aβ proteins are thought to play a dual role in central nervous system (CNS) in a concentration dependent manner. When secreted constitutively at picomolar concentration (Cirrito et al., 2003), these proteins increase neurogenesis (López-Toledano and Shelanski, 2004), enhance memory (Puzzo et al., 2008), reduce oxidative stress (Bishop and Robinson, 2004), and improve neuronal survival (Plant et al., 2003). However, pathological overproduction of the same protein at nanomolar concentration leads to neurotoxic effects. Aβ peptide in its native state is unstable in water, as one-third of its amino acids (-AA-) sequence is hydrophobic.

As a consequence, these peptides follow a hierarchy of aggregation from a monomer, through soluble oligomers including low molecular weight oligomer—paranuclei—high molecular weight oligomer—protofibrils; to a mature amyloid fibrilllary state relatively resistant to chemical denaturation and proteolytic digestion (Pallitto and Murphy, 2001; Glabe, 2008; Pryor et al., 2012).

Mounting evidence indicates that the overproduction of Aβ and its aggregation in the brain is a primary cause of AD (Jarrett et al., 1993; Hardy and Selkoe, 2002; Walsh and Selkoe, 2004; Lesné et al., 2006; Ferreira and Klein, 2011). Further, the anatomical observations in AD post-mortem brains and AD transgenic (Tg) mice revealed that Aβ secretion occurs mainly in the entorhinal cortex, hippocampus, temporal cortex, and frontoparietal cortex of the brain, the areas associated with learning, memory, and cognitive functions (Braak and Braak, 1991; Reddy and McWeeney, 2006). It is previously documented that β_(1−42)_ variant of amyloid is more prone to precipitation and aggregation than Aβ_(1−40)_, that further results into plaque formation (Lansbury, 1997; Yan and Wang, 2006). Further, Shankar and colleagues demonstrated that the insoluble amyloid plaque from AD brain cortex did not impair LTP unless solubilized to release Aβ dimers, suggesting that the plaque cores are largely inactive, but sequester Aβ dimers that are synaptotoxic (Shankar et al., 2008). In corroboration, studies indicate that early stage aggregates are likely to be more damaging compared to that of senile plaques (Pallitto and Murphy, 2001; Glabe, 2004; Pryor et al., 2012). Contrary to this, relatively weak correlation has been found between fibrillar plaque and severity of dementia in AD, as seen in some cognitively normal individuals with high amounts of deposited Aβ (Delaère et al., 1990; Dickson et al., 1995). However, the molecular basis underlying the progressive decrement of memory and cognitive functions in AD is not clear.

Aβ mediated dysregulation of N-methyl-D-aspartate (NMDA) receptors has been hypothesized as a potential mechanism in the pathophysiology of AD (Miguel-Hidalgo et al., 2002; Molnár et al., 2004; Snyder et al., 2005; Shankar et al., 2007; Malinow, 2012). Although, the role of ionotropic glutamate receptors in excitotoxic cell death associated with neurodegeneration is well-described (Doble, 1999; Miguel-Hidalgo et al., 2002; Fan and Raymond, 2007). The NMDA receptor antagonists are not suitable for long term treatment due to their unwanted side effects as they impair excitatory synaptic transmission, causing sedation, ataxia and memory loss (Nicoletti et al., 1996; Lee et al., 1999; Koller and Urwyler, 2010). Thus, it shifts the impetus of the research toward identifying a novel target which can modulate, and not mediate excitatory synaptic transmission in an attempt to obtain neuroprotective drugs with a good profile of safety and tolerability. More recently, the attention has shifted on to the role of metabotropic glutamate receptors (mGluRs) in Aβ oligomers mediated synaptic dysfunction (Renner et al., 2010). mGluRs are suggested to play a modulatory rather than direct role in excitatory glutamatergic synaptic transmission, making it a pharmacological avenue for producing modulatory action on glutamate systems in the CNS. mGluRs have been shown to play an important role in processes requiring synaptic plasticity, such as learning and memory, and neuronal development (Nakanishi, 1994; Zhong et al., 2000; Hannan et al., 2001; Wu et al., 2001). These also find implication in the pathophysiology of neurodegeneration (Kingston et al., 1999; Bruno et al., 2001).

Based on sequence homology and preferred signal transduction pathways, mGlu receptors have been divided into three subgroups comprising group I (mGlu1 and mGlu5), group II (mGlu2 and mGlu3) and group III (mGlu4, 6, 7, and 8) (Pin and Bockaert, 1995; Conn and Pin, 1997). This review focuses mainly on the mGluR5 subtype and its plausible role in the pathology of AD.

## Biology of mGluR5

mGluR5 is a transmembrane (TM) G-protein couple receptor positively coupled to phospholipase C via G_αq/11_ and inositol 1,4,5 trisphosphate formation (Alberts et al., 1994; Abdul-Ghani et al., 1996). Structural analysis shows a large extracellular amino-terminal domain with 17 cysteine's in conserved position (about 65 kDa, constituting one-half of the protein), 7TM hydrophobic segments and intracellular carboxy-terminal domain. It also contains an additional hydrophobic domain in the extracellular domain, postulated to form the ligand binding domain and the segments surrounding this region possible involved in G-protein coupling (Pin and Duvoisin, 1995). mGluR5 exist in three splice variant forms, mGlu5a, mGlu5b, and mGlu5d (Joly et al., 1995; Minakami et al., 1995; Romano et al., 1996; Malherbe et al., 2002). In the post-synaptic elements, mGlu5 receptors are physically linked to the NR2 subunit of NMDA receptors via a chain of interacting proteins, including PSD-95, Shank and Homer (Tu et al., 1999). mGluR5 activation increases intracellular calcium levels further stimulating protein kinase C activation. mGlu5 receptors primarily display a peri-synaptic localization at the post-synaptic membrane of glutamatergic neurons (Lujan et al., 1996), where they often regulate neuronal excitability by modulating currents through ionotropic glutamate receptor channels (Shigemoto et al., 1993, 1997). These are found to be localized in the neurons and glia throughout the CNS including the cortex and the hippocampus (Romano et al., 1995; Kerner et al., 1997; Biber et al., 1999).

## Pharmacology of mGluR5

Even though mGluRs were identified and cloned much earlier, the pharmacology of these receptors was not explored much for several years due to the lack of subtype selective antagonists. The very first mGluR5 selective agonist was CHPG, [(RS)-2-chloro-5-hydroxyphenylglycin], which showed the potentiation response in NMDA-induced depolarizations in rat hippocampal slices by selectively activating the mGluR_5a_ (Doherty et al., 1997). Shortly after, conformationally constrained cyclobutane analogs of quisqualic acid, (Z)- and (E)-CBQA were described as selective and highly potent mGlu5 receptor agonists (Littman et al., 1999). Using high throughput screening, Varney et al. (1999) reported the discovery of SIB-1757 and SIB-1893, the first subtype selective mGlu5 receptor antagonists. In rat striatal slices, SIB-1757 inhibited (S)-3,5-dihydroxyphenylglycine (DHPG) stimulated phosphoinositide hydrolysis (IC_50_ value 3.3 mM) but failed to inhibit it in the cerebellum where mGlu5 receptor expression is low. Subsequently, two more compounds MPEP [2-Methyl-6-(phenylethynyl)pyridine] and MTEP [3-((2-Methyl-4-thiazolyl)ethynyl)pyridine], were identified which showed improved selectivity against mGluR5 and were easily penetrable through blood brain barrier, therefore making them suitable for *in vivo* brain studies (Gasparini et al., 1999). Schield analysis suggested that these drugs reduce the efficacy of glutamate-stimulated phosphoinositide hydrolysis without affecting the EC_50_ of the glutamate and hence, behave as non-competitive antagonists (Schoepp et al., 1999).

### Pathological involvement and therapeutic potential in Alzheimer's disease

#### mGluR5 in neuronal cells

As mentioned previously, the Aβ oligomers (Aβo) were hypothesized to exert their synaptotoxic effects through NMDA receptors, which is known to be the early symptom of cognitive dysfunction in AD. Memantine, a non-competitive NMDA receptor antagonist, and an FDA approved drug for AD is known to slow down the progression of moderate-to-severe AD (Lipton, 2007). However, the complete NMDA receptor blockade may also lead to memory impairment. In current scenario, researchers and the pharmaceutical companies are investigating for the drug targets which can modulate, rather than mediate, the toxic effect of NMDA receptors. Further, the study by Song et al. (2008) reported that memantine failed to attenuate Aβ-induced potentiation of extracellular glutamate levels, suggesting the involvement of other surface receptors too. Since, mGluR5 is highly expressed in the brain cortex and hippocampal regions, it can be assumed that mGluR5 may have a predominant role in cognitive dysfunction related brain disorders like AD. Plenty of evidence suggests that the neuronal expression of mGluR5 is required for several physiological functions, in interaction with NMDA receptors; possibly via Homer/PSD95/Shank protein complex (Tu et al., 1999; Pisani et al., 2001; Collett and Collingridge, 2004; Homayoun et al., 2004; Matta et al., 2011; Won et al., 2012). However, while constitutive expression of mGluR5 is required for NMDA receptor mediated synaptic plasticity, pathologically overexpressed mGluR5 may possibly trigger the neurotoxic downstream signaling pathway that may further lead to cell death. In addition, pharmacological blockade or genetic alteration of mGluR5 is found to be neuroprotective (Bruno et al., 2000; Schiefer et al., 2004; Vernon et al., 2005; D'Antoni et al., 2011; Hamilton et al., 2014), further emphasize toward its contribution in the neurodegeneration. Further, it has also been shown that mGluR5 antagonists exhibit neuroprotection against excitotoxic degeneration (Kingston et al., 1999; O'Leary et al., 2000; Movsesyan et al., 2001) as well as β-amyloid-induced toxicity in cortical cultures (Bruno et al., 2000). Intriguingly, mGluR5 has been found to be upregulated in the cerebral cortex of the patients suffering from Down's syndrome (Oka and Takashima, 1999). Since the dementia associated with Down's syndrome shares a common amyloid pathology with AD (Weksler et al., 2013), the role of mGluR5 in AD pathogenesis can be as well speculated. Although limited evidence directly associates mGluR5 signaling with AD, several studies have shown potential mechanistic interactions between AD-associated molecules and mGluR5. Investigations by Renner et al. (2010) exposed a mechanism whereby membrane bound Aβo induced the abnormal accumulation and overstabilization of a glutamate receptor to synapses through direct or indirect interactions. Moreover, while mGluR5 gene transfer into the CA1 region resulted in neurodegeneration; downregulation or pharmacological blockage of mGluR5 in 2XTg_α-syn/APP_ mice and in neuronal cultures was protective against the neurotoxic effects of α-syn and Aβ (Overk et al., 2014). In corroboration, antagonists of mGluR5 prevented Aβo-induced dendritic spine loss and AD transgene learning and memory deficits (Um et al., 2013). A couple of studies also showed that Aβo from synthetic, cellular and human AD brain sources suppresses LTP and enhances LTD. These actions are mimicked by mGluR5 agonists and inhibited by mGluR5 antagonists (Wang et al., 2004; Shankar et al., 2008; Rammes et al., 2011; Hu et al., 2014). However, controversies over the mGluR5 acting as a receptor or co-receptor for Aβo remain unresolved. In a study, Lauren et al. (2009) demonstrated that Aβo could suppress LTP in hippocampal slices from normal mice, but not in hippocampal slices from mice lacking cellular prion protein (PrP^C^). In addition, many other studies also indicate that PrP^C^ is required for the actions of Aß to alter mGluR5 regulation of synaptic plasticity (Freir et al., 2011; Nicoll et al., 2013; Um et al., 2013; Hu et al., 2014; Hamilton et al., 2015), suggesting the role of mGluR5 as a co-receptor. Though, the high-affinity binding between Aβo and PrP^C^ may indicate a functional link between the two proteins, the involvement of other surface receptors cannot be ruled out as PrP ablation reduced the binding of Aβo to neurons by only 50% (Lauren et al., 2009). However, a successive study ruled out the PrP^C^ mediated oligomer induced memory impairment and cytotoxicity (Balducci et al., 2010). Concurrently, Kessels et al. (2010) also showed that Aβ_42_ blocked LTP irrespective of the presence of PrP^C^. Therefore, it becomes pertinent to critically evaluate the interaction of Aβo-PrP^C^-mGlu5R proteins and their involvement in synaptic dysfunction and memory loss.

### mGluR5 in non-neuronal cells

Glial cells constitute the majority of the brain cells and are emerging as major regulators of nervous system development, function, and health. Extensive investigations in the recent past have led to a better understanding of glial functions in health and disease, in view of their ability to not only support the neurons, but also defend and protect them from injury (Ransom and Sontheimer, 1992; Tsacopoulos and Magistretti, 1996; Verkhratsky et al., 1998; Boucsein et al., 2003; Rakic, 2003). Glial cells have the impressive ability to sense any neural injury and respond by undergoing “reactive gliosis,” a process whereby glia exhibit dramatic changes in morphology and gene expression patterns, migrate to the injury site, and manage brain responses to any damage. Reactive gliosis has been a major topic of study in the field of glial cell biology for over a decade, but molecular pathways mediating the pathological neuron-glia signaling have remained largely undefined.

Interestingly, mGlu5 receptors, like neurons are also expressed in non-neuronal cells, including microglia and astrocytes, where their activation exerts numerous effects that are crucial for glial cell function and glial-neuronal interaction under physiological and pathological conditions (Verkhratsky and Kettenmann, 1996; Verkhratsky et al., 1998). In healthy brain, microglia is known to illustrate defensive immune system (Zabel and Kirsch, 2013). However, in response to prolonged inflammatory stimulus under pathological conditions, the chronically activated microglia can lead to neurodegeneration by releasing a plethora of pro-inflammatory cytokines (Block et al., 2007; Holmes et al., 2009; Mosher and Wyss-Coray, 2014; Streit and Xue, 2014) and reactive oxygen species (Wilkinson and Landreth, 2006; Dewapriya et al., 2013). Consistent with several observations in neurodegenration, microglial activation was observed in brains of AD patients (McGeer et al., 1987; Shimohama et al., 2000) as well as in animals model of AD (Kim et al., 2014), suggesting the inflammatory role of these cells in the pathophysiology of AD. Moreover, Paranjape et al. (2012) described that small, soluble Aβ_42_ protofibrils induce much greater microglial activation than monomers or mature insoluble fibrils. Epidemiological studies also indicate that anti-inflammatory drugs may reduce AD incidence (McGeer and McGeer, 2007). However, clinical observations reported that the non-steroidal anti-inflammatory therapy did not slow down the cognitive decline associated with AD (Martin et al., 2008). Thus, preventing the prolonged microglial activation, rather than the counteracting the inflammation at later stages can be a better protective strategy in AD and other related neurodegenerative diseases. Although mGluR5 mRNA has been found to be expressed predominantly in microglial cultures (Biber et al., 1999; Byrnes et al., 2009), very few studies demonstrate its role in the microglial activation and neuroinflammation. The activation of microglia in AD can be speculated to be caused by Aβo in the extracellular compartment through the stimulation of mGlu5 receptors. Surprisingly, mGlu5 receptor was found to negatively regulate the release of microglia associated inflammatory factors and related neurotoxicity (Byrnes et al., 2009). Further, Liu et al. (2014) demonstrated that mGluR5 was involved in LPS-induced innate immune response in microglia. It has been shown that activated microglia can acquire either a neurotoxic M1 (proinflammatory), or a neuroprotective M2 (anti-inflammatory) phenotype (Kigerl et al., 2009; Liao et al., 2012; Cherry et al., 2014; Moehle and West, 2014; McGeer and McGeer, 2015) which are thought to be dependent on the intensity of insult (acute or chronic), type of stimuli (Aβ, TNF-β, IL1β or other toxic agents) and the stage of disease progression (early or late stage). Thus, investigating mGluR5 protein expression and their role in Aβ-induced microgliosis, will open new avenues in understanding the AD pathogenesis and possibly will provide potential therapeutic approach in the future.

Moreover, activated microglia can also potentially induce the activation of astrocytes (Liu et al., 2011). It was previously believed that astrogliosis appears in the late stages of AD. However, recent findings depict their involvement even in early stage of disease progression as reported in animal model of AD (Kuchibhotla et al., 2009; Verkhratsky et al., 2010; Yeh et al., 2011; Kulijewicz-Nawrot et al., 2012). Soluble Aβo may trigger astroglial remodeling that in turn might have indirect consequence on adjacent neurons. More recent studies demonstrated a direct implication of glial activation in the induction of neuronal death by Aβo (Abeti et al., 2011; Orellana et al., 2011). They showed that microglia instigates the Aβ mediated toxic effect by promoting the release of glutamate and ATP through glial cells that further leads to neuronal death. However, there is a missing link between the Aβ and the glial activation, understanding which can solve the complexity of AD pathology. mGlu5 receptors are expressed largely in cortical and hippocampal astrocytes (Biber et al., 1999). Recent studies showed that mGluR5 mRNA and the protein level are upregulated in cultured astrocytes following 2 days of treatment with Aβo (Casley et al., 2009; Lim et al., 2013), whereas pro-inflammatory agents, like TNF-α, IL-1β & LPS downregulate their expression level in astrocyte cultures (Aronica et al., 2005; Berger et al., 2012). In prospect of these findings, it can be assumed that astrocytes behave differently based on the type of stimuli received from the extracellular milieu, and the binding receptor. Indeed, very little is known about the functional consequences of elevated mGluR5 expression in the patho-mechanism of AD. Shrivastava et al. (2013), reported a strong enrichment of mGlu5 receptors on reactive astrocytes surrounding Aβ plaques. Furthermore, they also observed a rapid binding and clustering of Aβo over the astrocytic cell surface resulting in a diffusional trapping and clustering of mGlu5 receptors within Aβ clusters which in turn led to an increased ATP release. This observation further strengthens the emerging concept that mGluR5 play a major role in Aβ-mediated neurodegeneration and plausibly through the glial-neuronal interaction.

**Figure 1:**
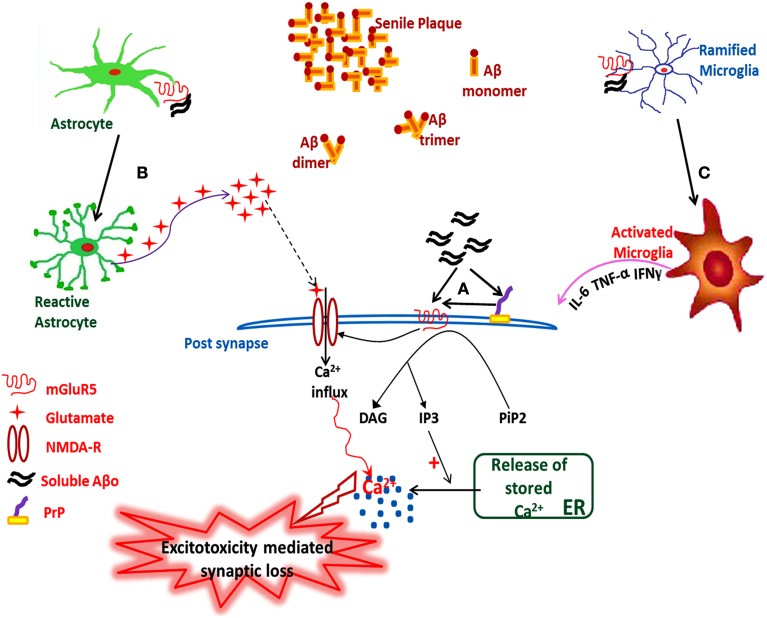
**Hypothetical illustration showing the sAβo-PrP^C^-mGluR5 interaction mediated pathological events in AD**. **(A)** Interaction of sAβo with mGluR5 either directly or through PrP at post-synaptic neurons, which either modulates the NMDA-R activity or triggers downstream second messenger cascades, **(B)** Astrocytic mGluR5 activation by sAβo generates sustained Ca^2+^ oscillations inside the reactive astrocytes which triggers the release of stored intracellular glutamate, thus enhancing the neuronal excitability leading to excitotoxicity followed by synaptic loss, **(C)** Interaction of sAβo to microglial mGluR5 triggers microglia activation followed by the release of proinflammatory cytokines leading to neuroinflammation. sAβo, soluble amyloid-β oligomer.

In summary, the interaction of Aβo with transmembrane mGluR5 might be the triggering factor for the pathogenesis of Alzheimer's disease, as depicted in Figure 1.

## Conclusion

Pathways of neurodegeneration in AD remain elusive, and so does the cure. Growing evidences hint toward Aβ mediated neurotoxicity to be central to the pathophysiology of the disease, possibly through the NMDA receptor dysfunction. However, targeting the ionotropic glutamate receptors also results in perturbation of other physiological processes linked with glutamatergic synaptic transmission, and hence presents as an ineffective treatment strategy. Consequently, it shifts the focus toward the metabotropic glutamate receptors, a family of G-protein coupled receptors, which can modulate, rather than block the glutamatergic pathways. Much of the observations on the role of mGluR5 using specific non-competitive antagonists like MPEP suggest the role of the receptor in certain physiological as well as pathological events along with the NMDA receptors. The ability of Aβo to actively interact with mGluR5 indicate the possible role of the receptor in neurodegeneration associated with Aβ proteinopathies. The neuroprotective effects of downregulation or pharmacological blockage of mGluR5 emphasize toward the potential of the therapeutic interventions targeting these receptors. Further, enhanced expression of mGluR5 observed in glial cells upon activation, its ability to curb inflammation and modulate immune response, along with its synergistic contribution in the Aβ mediated activation of glial cells renders it the status of potential candidate for delineating the neurodegenerative mechanisms as well as therapeutic interventions.

### Conflict of interest statement

The authors declare that the research was conducted in the absence of any commercial or financial relationships that could be construed as a potential conflict of interest.
